# Validation of Simple Prediction Equations for Step Count in Japanese Patients with Chronic Obstructive Pulmonary Disease

**DOI:** 10.3390/jcm11195535

**Published:** 2022-09-21

**Authors:** Yuichiro Azuma, Yoshiaki Minakata, Mai Kato, Masanori Tanaka, Yusuke Murakami, Seigo Sasaki, Kazumi Kawabe, Hideya Ono

**Affiliations:** 1 Department of Respiratory Medicine, National Hospital Organization Wakayama Hospital, Mihama-cho, Wakayama 644-0044, Japan; 2 Department of Respiratory Medicine, Hashimoto Municipal Hospital, Hashimoto-shi, Wakayama 648-0005, Japan

**Keywords:** chronic obstructive pulmonary disease, COPD, physical activity, step count, prediction equation

## Abstract

Physical activity is decreased in patients with chronic obstructive pulmonary disease, and decreased physical activity leads to a poor prognosis. To determine an individual’s target step count from the measured step counts and predicted step counts, simple and detailed prediction equations for step count were developed. To verify the validity of the simple prediction equation, the validity of the simple equation was evaluated in a different cohort and the correlation between the step counts calculated by the simple equation and those by the detailed prediction equation were evaluated. When the step counts calculated by the simple prediction equation for all participants were compared with the measured step counts, a significant correlation was obtained among them, and the calculated values were found to be reproducible with the measured values in patients with a measured step count of <6500 by Bland–Altman plots. Furthermore, the values calculated by the simple prediction equation and those calculated by the detailed prediction equation showed a significant correlation. In conclusion, the simple prediction equation was considered reasonable.

## 1. Introduction

Physical activity is decreased in patients with chronic obstructive pulmonary disease (COPD) compared to healthy controls [1,2], and decreased physical activity leads to a poor prognosis [3,4]. Physical inactivity is typically associated with all-cause mortality in patients with COPD [5].

Recently, the use of tri-axial accelerometers to evaluate physical activity has attracted attention, and various studies have been conducted [6,7,8,9,10,11,12,13,14]. However, tri-axial accelerometers are not yet widely available and are not familiar to general physicians. A pedometer is a simple device for evaluating physical activity, and the daily step count has been used as an indicator of physical activity [15,16,17]. For the general population, the American College of Sports Medicine recommends moderate-intensity cardiorespiratory exercise training for ≥30 min/day ≥5 days/week, vigorous-intensity cardiorespiratory exercise training for ≥20 min/day ≥3 days/week (≥75 min/week), or a combination of moderate- and vigorous-intensity exercise to achieve a total energy expenditure of ≥500–1000 metabolic equivalent (MET)·min/week [18]. However, there have been no recommendations regarding target values of physical activity for patients with COPD. Since patients with COPD have limited physical activity due to reduced lung function, goals comparable to those of healthy individuals may be excessive and should be recommended based on the number of steps expected for each COPD patient.

The factors related to the daily step count among anthropometry, dyspnea, and pulmonary function test findings in Japanese patients with COPD were evaluated and a simple prediction equation for the daily step count using three variables, namely age, a modified Medical Research Council (mMRC) dyspnea scale, and inspiratory capacity (IC), was developed [19]. In addition, since physical activity in COPD patients is considered to be affected by various factors, the use of more factors was examined and a detailed prediction equation for the step count using four variables were developed; these four variables are a 6-min walking distance (6MWD), the mMRC dyspnea scale, the Hospital Anxiety and Depression Scale (consisting of seven items for anxiety; HADS-A) and the forced expiratory volume in one second as a percentage of the predicted value (FEV1 %pred) [20]. Although the detailed prediction equation is considered more accurate, measuring the 6MWD and HADS-A is not easy in daily clinical practice. Therefore, the simple prediction equation may be more practical. Recently, a study has been started to try to increase the step count to reach the target value, which was determined from the measured step counts and predicted values of the step counts by the simple prediction equation [21]. It is necessary to verify whether the simple prediction equation is indeed accurate.

In the current study, to verify the validity of the simple prediction equation, the validity of the simple equation was evaluated in a different cohort and the correlation between the step count calculated by the simple equation and that by the detailed prediction equation was examined.

## 2. Materials and Methods

### 2.1. Design

The previous data from a multicenter, prospective cross-sectional study conducted at 23 institutions belonging to National Hospital Organization of Japan were used as secondary use, in which the detailed prediction equation of physical activity in Japanese patients with COPD was developed [20].

### 2.2. Evaluations

To verify the simple prediction equation of step count for the current cohort, the standard value of the step count for each patient by applying the simple equation was calculated and compared with the actual measured value. Moreover, the step counts calculated by the simple prediction equation was compared with those calculated by the detailed prediction equation and the correlation between the two was examined.

### 2.3. Measurement of Step Count

Subjects wore a tri-axial accelerometer (Active style Pro HJA-750C [HJA]; Omron Healthcare, Kyoto, Japan) on their waist for 15 to 29 days. From the measured days, the first and last days, rainy days, holidays, days with an average temperature of less than 2.5 °C, days with unusual activities, and days with <8 h per day of measurement time were excluded. Of the remaining valid days, the average of the data from the first three days was used.

### 2.4. Prediction Equations

The equation by Nakanishi et al. was employed as a simple prediction equation [19]. This is the equation created using a multiple regression analysis with the age, gender, body mass index (BMI), smoking history, IC, forced vital capacity as a percentage of the predicted value (FVC %pred), FEV1 %pred, and mMRC dyspnea scale as independent variables. The simple equation was as follows:

**step count = (−0.079 × age–1.595 × mMRC dyspnea scale + 2.078 × IC + 18.149)^3^**

The equation by Minakata et al. was used as a detailed prediction equation [20]. This is the equation created based on a multiple regression analysis with the age, gender, height, weight, BMI, smoking history, IC, FVC %pred, FEV1/FVC, FEV1 %pred, 6MWD, lowest percutaneous oxygen saturation during the 6MW test, upper arm circumference, subcutaneous fat thickness of triceps branch, grip strength, fasting blood glucose, hemoglobin A1c, red blood cells, hemoglobin, brain natriuretic peptide, albumin, HADS-A, mMRC dyspnea scale, treatment history, rehabilitation history, and comorbidities as independent variables. The detailed equation was as follows:

**step count = (0.01 × 6MWD–0.666 × mMRC dyspnea scale + 0.155 × HADS-A + 0.029 × FEV1 %pred + 9.843)^3^**

### 2.5. Statistical Analyses

Statistical analyses were performed using the GraphPad Prism 7 software program (GraphPad Software, San Diego, CA, USA). Correlation coefficients and Bland–Altman plots were used to compare the measured and calculated step counts. The level of statistical significance was considered to be *p* < 0.05.

### 2.6. Ethical Consideration

This study was conducted in accordance with the Declaration of Helsinki and was approved by the local ethics committee (IRB Committee of National Hospital Organization Wakayama Hospital; approval number: 03-4; approval date: 22 April 2021) and has been registered with the University Hospital Medical Information Network (UMIN 000047281, 26 March 2022). The contents of this study and the opportunity to reject the agreement were explained on the website of the National Hospital Organization Wakayama Hospital.

## 3. Results

**Figure 1:**
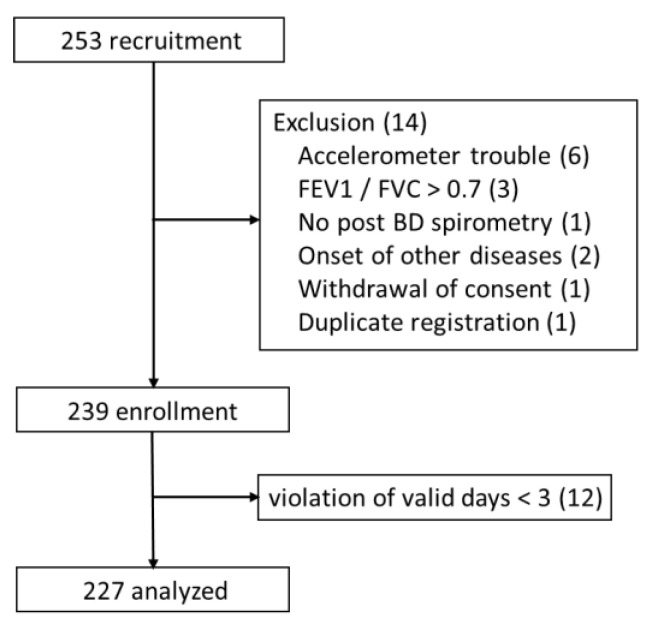
CONSORT diagram. Abbreviations: FEV1, forced expiratory volume in one second; FVC, forced vital capacity; BD, bronchodilator.

**Figure 2:**
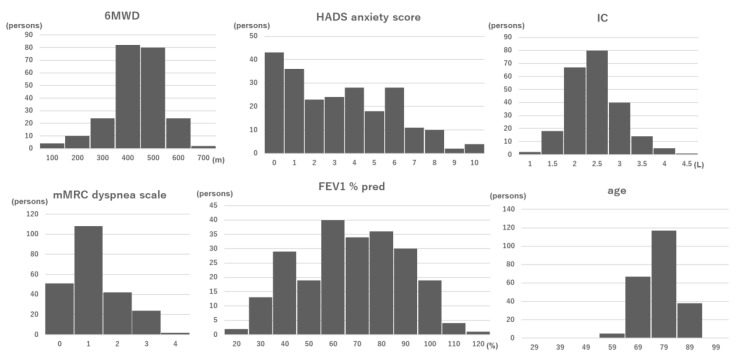
Histograms of the indicators. Abbreviations: 6MWD, 6 min walking distance; HADS, Hospital Anxiety and Depression Scale; IC, inspiratory capacity; mMRC, modified British Medical Research Council; FEV1 %pred, forced expiratory volume in one second % of predicted.

In all, 253 patients were recruited, and 239 were enrolled. Among enrolled patients, 12 had fewer than 3 valid days, so 227 patients were ultimately included in the analysis (Figure 1). The age was 73.1 ± 6.7 years old, the FEV1 %pred was 62.7% ± 20.9%, and the FVC %pred was 99.6% ± 19.5% (Table 1). Histograms for the indicators used in the prediction equations (6MWD, HADs anxiety score, IC, mMRC dyspnea scale, FEV1 %pred and age) are shown in Figure 2.

**Figure 3:**
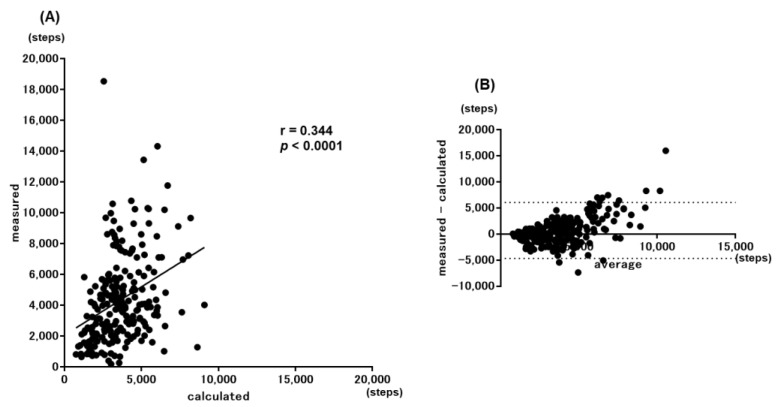
Correlations between the measured values and calculated values by the simple equation. (**A**) Scatter plot and (**B**) Bland–Altman plot.

**Figure 4:**
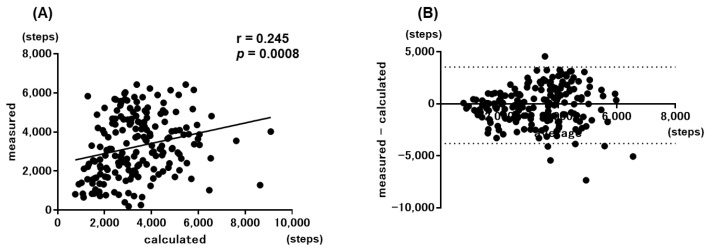
Correlations between the measured values and calculated values by the simple equation in patients with <6500 measured step. (**A**) Scatter plot and (**B**) Bland–Altman plot.

When the step counts calculated by the simple prediction equation for all 227 participants were compared with the measured step counts, a significant correlation was obtained among them (r = 0.344, *p* < 0.0001) (Figure 3A). However, when both step counts were examined with the Bland–Altman plots, there was no fixed bias, although a proportional bias was noted (Figure 3B). According to Nakanishi’s report, the values calculated by the simple prediction equation were reproducible with the values measured by the Bland–Altman plots in patients whose measured step count was <6500 steps [19]. In the current population, the calculated values by the simple prediction were also found to be reproducible with the measured values in patients with a measured step count <6500 (Figure 4). Furthermore, reproducibility was obtained in patients with a measured step count <7500.

**Figure 5:**
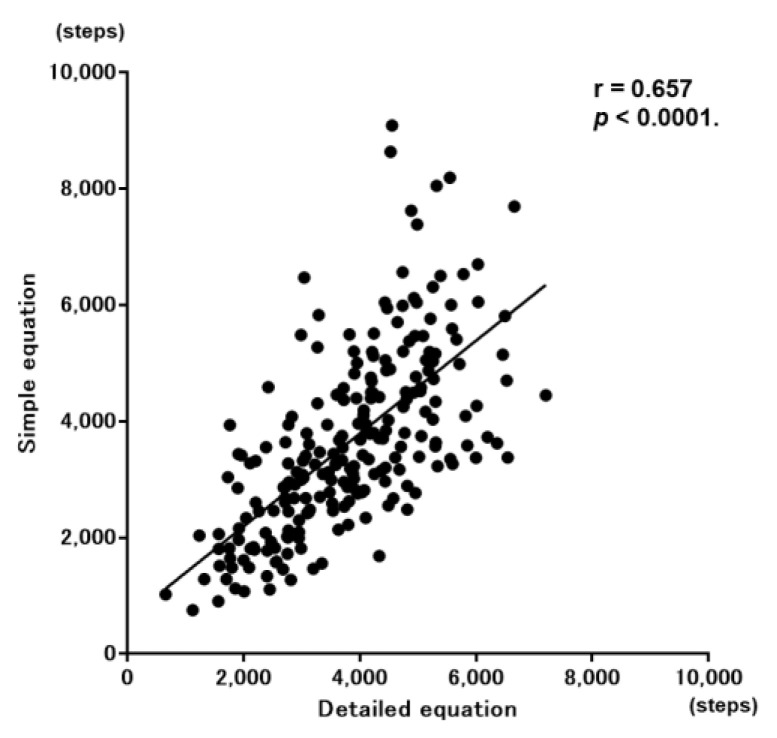
Correlation between the step count calculated by the simple prediction equation and that calculated by the detailed prediction equation.

The values calculated by Nakanishi’s simple prediction equation and those calculated by Minakata’s detailed prediction equation showed a significant correlation (r = 0.657, *p* < 0.0001) (Figure 5).

## 4. Discussion

The calculated step counts obtained from Nakanishi’s simple prediction equation showed a significant correlation with the measured step counts in the current population among patients with a measured step count <6500, confirming its reproducibility. In addition, it showed a significant correlation with the calculated step counts obtained from Minakata’s detailed prediction equation.

The simple prediction equation was confirmed to be reliable in patients with COPD and a low step count. The number of patients with a step count <6500 was 185 among 227 recruited patients (81.5%) in the current cohort and 121 among the 162 (74.7%) in the cohort for the development of Nakanishi’s simple prediction equation [19]. In the previous meta-analysis on the step counts in patients with COPD, the mean step count was 4579.3 (95% confidence interval 4310.2–5208.4) [22], which suggests that the daily step count of most patients with COPD is <6500. Therefore, Nakanishi’s prediction equation was found to be reproducible in different cohorts and may be a useful tool for evaluating the individual predictive step counts in most patients with COPD.

The step counts calculated by the simple prediction equation correlated with those calculated by the detailed prediction equation. The simple equation consisted of the age, mMRC dyspnea scale, and IC, whereas the detailed equation consisted of the 6MWD, mMRC dyspnea scale, HADS-A, and FEV1 %pred. The mMRC dyspnea scale was the only common variable between these two equations. The mMRC dyspnea scale has been reported to correlate with the step count and physical activity in many reports [15,23,24] and detailed prediction equations, and it was considered to be an important factor for the step count. Exercise capacity may be related to physical activity [1,2,25,26,27,28], and the 6MWD was included in the detailed equation. However, it was not included in the simple equation because exercise capacity was not evaluated as an independent variable for that equation. Exercise capacity has been reported to be more closely related to IC than to FEV1 %pred [29]. In the current cohort, the 6MWD was multicollinear with IC (r = 0.270, *p* < 0.001), as was FEV1 %pred (r = 0.458, *p* < 0.001) [20]. A decrease in the 6MWD can be predicted by a decrease in the IC/TLC ratio, with a 0.1-unit decrease in the baseline IC/TLC ratio resulting in a 12.7-m decrease in the 6MWD per year [30]. In addition, IC reflects the resting hyperinflation of the lung, and both resting and dynamic hyperinflation contribute to reduced physical activity in COPD [31], which suggests that a lower IC may lead to lower physical activity levels. The 6MWD and FEV1 %pred may thus be able to predict the step count relatively accurately, but IC might be used to some extent instead of them. Although the detailed equation is based on a larger number of variables and seems to be more accurate, the simple equation is easier to use in daily clinical practice.

The attempt to develop a method for setting individual target values of step counts based on the standardized values calculated by the simple prediction equation and the actual measured values is being made. A pilot study in which target values were provided showed the potential to increase the target achievement rate and step count when supplying such values [21]. The efficacy of providing a target value for the step count needs to be verified in future studies with a larger scale.

### Limitations

Several limitations associated with the present study warrant mention. First, most of the enrolled patients were men (93.8%). This distribution may be due to the fact that the rate of men with COPD is higher in Japan than in Western countries [32,33,34,35], and patients with bronchial asthma, which is thought to be relatively common in women, were excluded from the current cohort. While the number of women was small, gender was included in the regression analysis as an independent variable in both the simple and detailed prediction equations and was not extracted as a factor related to the step count. Further studies including a cohort in which more women participate might be required. Second, there might have been other step-related factors that were not employed as independent variables for the prediction equations. Ichinose et al. reported that the job status (employment) as well as mMRC dyspnea scale were factors related to the step count [24]. Further studies on other influencing factors not used in the current study should thus be conducted. Third, both the simple and detailed prediction equations were based on the Japanese population. Individual studies may be necessary for populations of other countries.

## 5. Conclusions

The simple equation for predicting the step count of Japanese patients with COPD was validated in different cohorts, and the values calculated by it were significantly correlated with those determined by the detailed prediction equation. The simple prediction equation was considered reasonable.

## Figures and Tables

**Table 1:** Patient characteristics.

Gender (Male/Female)	213/14
Age	73.1 ± 6.7
Smoking (pack-year)	64.2 ± 66.5
BMI	22.5 ± 3.4
COPD stage (1/2/3/4)	54/110/48/15
mMRC (0/1/2/3/4)	51/108/42/24/2
IC (L)	2.22 ± 0.56
FVC (L)	3.28 ± 0.78
FVC %pred (%)	99.6 ± 19.5
FEV1 (L)	1.64 ± 0.60
FEV1 %pred (%)	62.7 ± 20.9
FEV1/FVC (%)	49.7 ± 13.2
HADS anxiety score	3.3 ± 2.6
HADS depression score	4.3 ± 3.1

Abbreviations: BMI, body mass index; mMRC, modified British Medical Research Council; IC, inspiratory capacity; FVC, forced vital capacity; FVC %pred, FVC % of predicted; FEV1, forced expiratory volume in one second; FEV1 %pred, FEV1 % of predicted; HADS, Hospital Anxiety and Depression Scale; COPD, chronic obstructive pulmonary disease.

## Data Availability

The data that support the findings of this study are available from the corresponding author upon reasonable request.
